# Inactivation of Prostaglandin E_2_ as a Mechanism for UGT2B17-Mediated Adverse Effects in Chronic Lymphocytic Leukemia

**DOI:** 10.3389/fonc.2019.00606

**Published:** 2019-07-04

**Authors:** Eric P. Allain, Michèle Rouleau, Trang Le, Katrina Vanura, Lyne Villeneuve, Patrick Caron, Véronique Turcotte, Eric Lévesque, Chantal Guillemette

**Affiliations:** ^1^Pharmacogenomics Laboratory, Faculty of Pharmacy, Centre Hospitalier Universitaire de Québec (CHU de Québec) Research Center – Université Laval, Laval University, Québec City, QC, Canada; ^2^Division of Hematology and Hemostaseology, Department of Medicine I, Medical University of Vienna, Vienna, Austria; ^3^Division of Hemato-Oncology, Faculty of Medicine, CHU de Québec Research Centre – Université Laval, Laval University, Québec City, QC, Canada; ^4^Canada Research Chair in Pharmacogenomics, Québec City, QC, Canada

**Keywords:** UGT2B17, CLL, prostaglandins, glucuronidation, PGE_2_

## Abstract

High expression of the metabolic enzyme UDP-glucuronosyltransferase UGT2B17 in chronic lymphocytic leukemia (CLL) cells was associated with poor prognosis in two independent studies. However, the underlying mechanism remains unknown. We hypothesized that UGT2B17 impacts intracellular levels of hormone-like signaling molecules involved in the regulation of gene expression in leukemic cells. We initially confirmed in a third cohort of 291 CLL patients that those with high UGT2B17 displayed poor prognosis (hazard ratio of 2.31, *P* = 0.015). Consistent with the unfavorable prognostic significance of elevated UGT2B17 expression in CLL patients, high UGT2B17 expression was associated with enhanced proliferation of MEC1 and JVM2 malignant B-cell models. Transcriptomic analyses revealed that high UGT2B17 was linked to a significant alteration of genes related to prostaglandin E2 (PGE_2_) and to its precursor arachidonic acid, both in cell models and a cohort of 448 CLL patients. In functional assays, PGE_2_ emerged as a negative regulator of apoptosis in CLL patients and proliferation in cells models, whereas its effect was partially abrogated by high UGT2B17 expression in MEC1 and JVM2 cells. Enzymatic assays and mass-spectrometry analyses established that the UGT2B17 enzyme inactivates PGE_2_ by its conjugation to glucuronic acid (GlcA) leading to the formation of two glucuronide (G) derivatives. High UGT2B17 expression was further associated with a proficient inactivation of PGE_2_ to PGE_2_-G in CLL patient cells and cell models. We conclude that UGT2B17-dependent PGE_2_ glucuronidation impairs anti-oncogenic PGE_2_ effects in leukemic cells, thereby partially contributing to disease progression in high UGT2B17 CLL patients.

## Introduction

Chronic lymphocytic leukemia (CLL) is the most common form of adult leukemia in the western world (1). It is characterized by an accumulation of mature B-cells in peripheral blood, bone marrow, and other lymphoid organs. CLL has a highly variable course and generally develops at a slower pace than other leukemia subtypes. The accumulation of CLL cells in patients can be explained, in part, by defects in the regulation of apoptosis (2), but other studies have also shown evidence of clonal evolution (3, 4) and the importance of active proliferation in progressive disease (5, 6).

A significant increase in therapeutic options for CLL patients has been associated with improved survival. However, CLL remains incurable (7). Recent investigations have yielded insights into molecular indicators used to better predict clinical outcome of this disease. Of those, uridine diphospho-glucuronosyltransferase 2B17 (*UGT2B17*) expression was identified as a novel molecular marker for CLL progression in two independent CLL cohorts (8, 9). The initial report associated high *UGT2B17* mRNA expression with poor prognosis, unmutated-IGHV and other expression-based markers, such as LPL and CD38 (9). The second study reported high *UGT2B17* expression as a prognostic marker particularly for mutated-IGHV individuals, a subgroup of patients for which few indicators of progression currently exist (8). The *UGT2B17* gene encodes a member of a superfamily of metabolic enzymes responsible for the conjugation of small lipophilic substrates to glucuronic acid (GlcA) derived from its co-substrate UDP-GlcA (10). Glucuronidation inactivates the enzyme's substrates, increases their polarity and facilitates their elimination through bile or urine. The function of this metabolic route is to maintain homeostasis of endogenous molecules while protecting cells from potentially harmful chemicals arising from exogenous sources, including pharmacological compounds (11).

The underlying mechanism(s) by which the UGT2B17 protein may affect CLL malignancy and disease progression in patients remains unknown. Amongst the 19 human UGT isoforms, UGT2B17 is the only significantly expressed UGT in CLL cells. The UGT2B17 protein is enzymatically functional, as it was shown to conjugate UGT2B17 substrates such as androgens in cells isolated from CLL patients (9). It is plausible that UGT2B17 influences intracellular levels of hormone-like signaling molecules involved in the regulation of gene expression, with subsequent impacts on cancer cell growth and survival. We hypothesized that overexpression of UGT2B17 perturbs the bioavailability and response to endogenous B-cell modulators with consequences on the transcriptome of leukemic cells and neoplastic behavior. We report that high UGT2B17 modifies expression of genes related to prostanoids. It also impairs prostaglandin-mediated growth inhibition of malignant cells through direct inactivation of these hormone-like molecules by glucuronidation.

## Materials and Methods

### Patients, Cell Lines, and Culture

The B-cell neoplastic cell lines MEC1 and JVM2 were purchased from DSMZ (Braunschweig, Germany) in 2010 and ATCC (Manassas, VA) in 2015, respectively. The MEC1-2B17 and JVM2-2B17 cell models were generated by electroporation of parental cells with a pcDNA6 vector containing the coding sequence of UGT2B17 for stable overexpression. Electroporation was achieved with the Neon Transfection System (Thermo Fisher scientific, Waltham, MA). The JVM2-CTRL cells were produced by electroporation with the pcDNA6 vector and the MEC1-CTRL cells were prepared as described (9). All cell lines were cultured in RPMI medium supplemented with 10% fetal bovine serum (FBS), 1% penicillin/streptomycin, 1% sodium pyruvate and 1% L-glutamine. Selection of MEC1-2B17 cells was achieved by supplementing culture medium with 20 μg/mL blasticidin for 3 weeks, after which selection was maintained by growing cells with 10 μg/mL of blasticidin. MEC1-CTRL cells were supplemented with 3 μg/mL of puromycin. For JVM2-CTRL and JVM2-2B17 cells, selection for 3 weeks and subsequent growth was achieved by supplementing medium with 5 μg/mL blasticidin. All cell culture components were purchased from Wisent Bioproducts (St-Bruno, QC). Cells were regularly tested for mycoplasma, with the most recent carried out on February 9th, 2019.

Cryopreserved peripheral blood mononuclear cells (PBMCs) from 15 CLL patients diagnosed between 1987 and 2011 at Vienna General Hospital were used (Table S1). Cell purity was assessed by measuring CD5 and CD19 surface expression by cytometry prior to experimentation, with CLL cells representing 67% of total PBMCs on average. Primary cells from CLL patients were cultured in RPMI supplemented with 10% FBS, 1% sodium pyruvate, and 1% L-glutamine without antibiotics. All subjects gave written informed consent in accordance with the Helsinki Declaration and the study was evaluated and approved by local Ethical Research Committees of the Medical University of Vienna (Ethics vote 1499/2015) and the Center Hospitalier Universitaire (CHU) de Québec (A14-10-1205).

### Immunoblotting

For western blot, microsomal fractions were prepared from MEC1 and JVM2 by resuspending 10^8^ cells in 1 mL microsome buffer (4 mM potassium phosphate, 20% glycerol, pH 7.0). Cells were sonicated three times for 30 s, alternating with 30-s pauses on ice. Extracts were centrifuged twice at 12,000 × g for 22 min at 4°C. Supernatants were centrifuged at 105,000 × g for 60 min at 4°C to isolate the microsomal fraction that was resuspended in microsome buffer containing 0.5 mM dithiothreitol (DTT). Microsomal fractions (20 μg) were mixed with Laemmli sample buffer (Bio-Rad, Mississauga, ON), heated at 95°C for 5 min prior to SDS-PAGE and transfered to nitrocellulose membranes. Protein detection was adapted from a previously described immunoblotting strategy (12) using the polyclonal anti-UGT2B antibody EL-93 (1:2,000) for detection of UGT2B17 (13) or an anti-calnexin antibody (1:2,000, Enzo Life Science, Farmingdale, NY) as a loading control.

### Glucuronidation Assays

Enzyme activity assays, incubated at 37°C for 120 min, were conducted with cell homogenates (100 μg) prepared by resuspending 10^8^ JVM2 and MEC1 cells in 1 mL PBS containing 0.5 mM DTT or with microsomal fractions (20 μg) from primary cells from CLL patients (prepared as described above) in a final volume of 100 μL. The reaction assays also contained 50 mM Tris-HCl (pH 7.5), 10 mM MgCl_2_, 5 μg/mL pepstatin, 0.5 μg/mL leupeptin, 0.5 mM UDP-GlcA, 20 μg/mL alamethicin, and indicated substrates. Final substrate concentrations were 200 μM for prostaglandins (PG) and vorinostat (SAHA; Sigma-Aldrich) or 25 μM for dihydrotestosterone (DHT), testosterone (Testo) and estradiol (E_2_) (all from Steraloids, Newport, RI). Steroid glucuronides (-G) were analyzed by liquid chromatography and tandem mass spectrometry (LC-MS/MS) as previously described (14). For PGs, chromatographic separation was performed onto an ACE C18 HL column (3 μM packing material, 100 × 4.6 mm; Canadian Life Science, Peterborough, ON) and a Nexera system (Shimadzu Scientific instruments, Columbia, MD). Elution of PGs-G was done in isocratic conditions consisting of 65% methanol, 35% H_2_O, 1 mM ammonium formate at a constant flow rate of 0.9 ml/min. For vorinostat, chromatographic separation was performed onto an ACE C18 HL column (3 μM packing material, 100 × 4.6 mm; Canadian Life Science). The mobile phases were solvent A: H_2_O, 0.1% formic acid and solvent B: acetonitrile, 0.1% formic acid. Vorinostat-G was eluted using the following program: 0–1.5 min, isocratic 40% B; 1.5–1.6 min, linear gradient 40–90% B; 1.6–2.9 min, isocratic 90% B; 2.9-3.0 min, linear gradient 90–40% B; 3.1–6.0 min, isocratic 40% B. Relative amounts of glucuronides were compared by area under the curve (AUC) by tandem MS (API 6500; Sciex, Concord, ON) operated in multiple reactions monitoring mode (MRM) and equipped with a turbo ion-spray source. The following mass ion transitions (m/z) were used: 527.2 → 175 for PGE_2_-G and 441.3 → 265.1 for vorinostat-G. Both systems were controlled through Analyst Software (version 1.6.1, AB Sciex).

### Gene Expression Analysis

Publicly available data sets were used for analysis of overall survival (OS) of CLL patients and CLL expression profiles in relation to *UGT2B17* levels using the affy (v1.48) and limma (v3.26.9) packages for R (http://www.bioconductor.org). The first dataset was obtained from the International Cancer Genome Consortium (ICGC-CLLE-ES, *n* = 291) (15, 16). A second dataset was from the Gene Expression Omnibus (GEO-GSE13159, *n* = 448 untreated patients) (17). For MEC1 and JVM2 cell models, total RNA from three biological replicates was extracted using RNeasy mini kit, as per manufacturer's instructions (Qiagen, Toronto, ON). Samples were subjected to ribosomal RNA depletion before Illumina HiSeq2000 paired-end sequencing at Genome Québec McGill University and at the CHU de Québec Research center - Université Laval. Raw data was processed using the MUGQIC pipeline version 1.3. Briefly, reads were quality trimmed and aligned to the hg38 human genome. Differential gene expression analysis was performed using the edgeR and DESeq2 tools for R v3.2.2 (18, 19). Differences in gene expression were considered significant if Benjamini-Hochberg adjusted *P*-values for both tools were below 0.05. Analysis of enriched biological pathways was carried out using g:profiler (20) with the Reactome (21), KEGG (22–24), and GO biological process databases. Reactome FI plugin for Cytoscape v3.2.1 was also used for clustering of genes into modules and visualization of enriched pathways (25). Altered expression of selected genes was validated by quantitative real time PCR. Briefly, total RNA was DNase I-treated and purified using the RNeasy MinElute Cleanup kit (Qiagen, Hilden, DE) following the manufacturer's instructions. First-strand cDNA synthesis was accomplished using Superscript IV RNase H-RT (Invitrogen Life Technologies, Burlington, ON, CA), PCR purification kit (Qiagen, Hilden, DE) was used to purify cDNA. Oligoprimer pairs were designed by GeneTool 2.0 software (Biotools Inc, Edmonton, AB, CA), their specificity was verified by BLAST alignment to human RefSeq sequences and were synthesized by IDT (Integrated DNA Technology, Coralville, IA, USA) (Table S2). RT-qPCR quantification was carried out using the LightCycler 480 (Roche Diagnostics, Mannheim, DE). Reagent LightCycler 480 SYBRGreen I Master (Roche Diagnostics, Indianapolis, IN, USA) was used as described by the manufacturer with 2% DMSO. Relative quantity was calculated using the second derivative method and by applying the delta Ct method (26). *B2M, HPRT1*, and *UBC* were used as reference genes for normalization (27). Quantitative qPCR measurements were performed in compliance with MIQE guidelines by the Gene Expression Platform of our institution (28, 29).

### Cell Proliferation and Viability Assays

Cells were plated at 1 × 10^4^ cells/well (MEC1) or 5 × 10^4^ cells/well (JVM2) in 96-well U-bottom tissue culture plates (BD Bioscience, Mississauga, ON). For treatment assays, growth media was supplemented with PGs or vehicle (ethanol) at time of plating and renewed every 48 h. PGE_2_, Butaprost and PGE_1_-OH, used at concentrations indicated in the text, were purchased from Cayman chemicals (Ann Arbor, MI). Every 24 h, an aliquot of cells was stained with trypan blue (50%) and counted with a TC-10 automated cell counter (Bio-Rad). Assays were replicated at least three times in duplicate. For MTS assays, 20 μL of CellTiter aqueous one solution cell proliferation reagent (MTS Promega, Madison, WI, USA) was added to 100 μL of cells. Absorbance at 490 nm was read after a 4-h incubation at 37°C. To further assay cell proliferation, cells were labeled with 5 μM of CFSE (Thermo Fisher Scientific) and incubated at 37°C for 10 min before rinsing once with DPBS and plating at 4 × 10^6^ cells/mL in 12-well culture plates with whole medium 72 h prior to analysis.

### Migration Assays

CLL PBMCs were plated at 3 × 10^6^ cells/mL in 24-well culture plates 24 h prior to migration assays. Cells were then treated for 24 h with PGE_2_ (1–5 μM) before initiation of migration experiments. Transwell 5 μm-pore inserts (Corning, New-York, NY) were then placed in a 24-well plate with 600 μL of medium in the lower well and 100 μL in the upper well, containing 5 × 10^5^ CLL cells. Cells were treated with either vehicle (PBS) or CXCL12 (200 ng/mL) before incubation for 4 h at 37°C. Cells in the upper and lower compartments were recovered in separate tubes by incubation with a non-enzymatic cell dissociation solution (Sigma-Aldrich, Oakville, ON) for 10 min at 37°C. Each tube was then spiked with 50 μL of 123eBeads (Thermo Fisher Scientific) and counted using a FACS Canto II flow cytometer (BD Bioscience). Migration was calculated with the following formula:

$$\begin{array}{l} \%\;\text{Migration}=\;\frac{Cells\;in\;lower\;chamber}{Cells\;in\;lower\;chamber\;+\;Cells\;in\;upper\;chamber}\;\times 100 \end{array}$$

### Flow Cytometry Analyses

Each patient PBMC sample was thawed and diluted to 3 × 10^6^ cells/ml prior to identification of CLL cells by analysis of cell surface markers using PerCPCy5.5-conjugated anti-human CD5 (BD Pharmingen), PE-Cy7-conjugated anti-human CD19 (BD Bioscience) and APC-Cy7-conjugated anti-human CD45 (BD Bioscience). All analyses were conducted with a FACS Canto II flow cytometer (BD Bioscience).

### Cell Death

Aliquots of 5 × 10^5^ cells were centrifuged at 520 × g and rinsed twice with Dulbecco's Phosphate Buffered Saline (DPBS) before resuspension in Annexin V binding buffer (50 mM HEPES, 700 mM NaCl, 12.5 mM CaCl_2_, pH 7.4). Cells were labeled with Alexa Fluor 647-conjugated Annexin V as per manufacturer's instructions (Thermo Fisher scientific) and propidium iodide (PI; 4 ng/mL) in the dark, on ice, for 30 min prior to analysis.

### BCR Stimulation Assays

Primary CLL PBMCs (5 × 10^5^ cells) were treated with PGE_2_ (1–5 μM) or DMSO 2 h after thawing. Cells were primed with PMA (10 ng/mL, Sigma-Aldrich) for 1 h, then stimulated with LPS (200 ng/mL Sigma-Aldrich) or *F*(ab')_2_ anti-human IgM (10 μg/mL, Southern Bioscience, Birmingham, AL). For assessment of ERK phosphorylation, cell aliquots (5 × 10^5^) were labeled with LIVE/DEAD® fixable dead cell stain kit (Thermo Fisher Scientific) as per manufacturer's instructions after 15 min of stimulation, then fixed with 3.7% paraformaldehyde and permeabilized with 90% ice-cold methanol before staining with 5 μL APC-conjugated rabbit anti-human p44/42 MAPK (pERK1/2, Cell Signaling Technologies, Danvers, MA) and analysis. For assessment by surface markers, aliquots of 5 × 10^5^ cells were labeled with LIVE/DEAD® 24 h post-stimulation, rinsed once with DPBS and incubated 30 min on ice with mouse anti-human PE-conjugated CD80 (BioLegend, San Diego, CA) and APC-conjugated CD86 (BD Pharmingen) before analysis.

### Statistical Analysis

The half maximal inhibitory concentrations (IC_50_) were calculated by fitting variable slope non-linear curves to normalized response data from PGE_2_ treatments. Statistical analysis was carried out using a two-tailed Student's *t*-test, unless otherwise indicated in the legends. Analysis of overall survival was done using the Kaplan-Meier method and the Log-Rank test. Statistics were performed using GraphPad Prism v5 (GraphPad Software Inc., La Jolla, CA) and R v3.2.2. Statistical significance is defined as ^*^ *P* < 0.05, ^**^ *P* < 0.01. Each experiment was performed with three biological replicates, unless otherwise indicated in figure legends.

## Results

### UGT2B17 Confers a Proliferative Advantage to Malignant B- Cell Models

**Figure 1:**
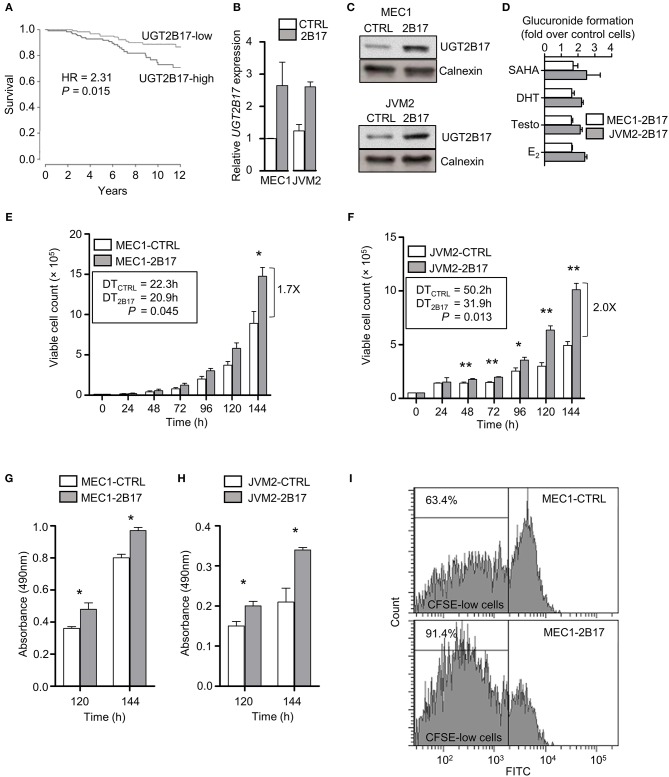
UGT2B17 confers a proliferative advantage to leukemic cells. **(A)** High *UGT2B17* levels are significantly associated with reduced overall survival of CLL patients. Kaplan Meier plot represents survival of 291 CLL patients of the ICGC cohort, dichotomized according to the median expression of *UGT2B17*. **(B–D)** The malignant B-cell models MEC1 and JVM2 stably expressing low (CTRL) or high UGT2B17 (2B17) levels, as characterized **(B)** at the mRNA level by qPCR, **(C)** at the protein level by immunoblotting of the microsomal fraction with a UGT2B-specific antibody; and **(D)** by measuring the glucuronidation activity in cell homogenates using the UGT2B17 substrates vorinostat (SAHA), testosterone (Testo), dihydrotestosterone (DHT), and estradiol (E_2_). Calnexin was used as a protein loading control in **(C)**. **(E–F)** High UGT2B17 expression enhances the proliferation rate of **(E)** MEC1 and **(F)** JVM2 neoplastic cells. Viable cell count was determined every 24 h for 7 days. Doubling times (DT) were calculated from the exponential growth phase (96–144 h). MTS assays of **(G)** MEC1 and **(H)** JVM2 cells also indicated increased proliferation of UGT2B17 overexpressing cells. **(I)** Representative cytometry data of CFSE labeling in MEC1 72 h after plating. CFSE-low cells represent the fraction of cells having undergone at least one cell division. Data represent mean ± SEM of three independent experiments. Cellular phenotypes associated with high UGT2B17 were reproducibly observed over time, attesting of the stability of cell models. ^*^ *P* < 0.05, ^**^ *P* < 0.01. Comparisons without reported *P*-value are non-significant.

A significant reduction in overall survival (OS) was established in 291 CLL patients from the International Cancer Genome Consortium (ICGC) expressing high *UGT2B17* levels (Figure 1A). Neoplastic B-cell models MEC1 and JVM2 stably overexpressing UGT2B17 were created to examine its role in CLL progression. These two cell lines were chosen on the basis of relatedness to CLL and diversity of cytogenetic aberrations. Overexpression was confirmed at the mRNA (2.6-fold) and protein (2.6-fold) levels in the MEC1-2B17 cells, and the functionality of the enzyme was supported by an enhanced glucuronidation activity for characteristic UGT2B17 substrates vorinostat (SAHA), testosterone (Testo), dihydrotestosterone (DHT), and estradiol (E_2_) by 1.6–1.7-fold (*P* < 0.05), compared to control cells levels (Figures 1B–D). In the JVM2-2B17 model, a 2.5-fold enhanced mRNA expression and 2.2-fold higher protein levels were observed with a corresponding 2.1–2.5-fold enhanced UGT2B17 activity (*P* < 0.05) (Figures 1B–D). Consistent with the unfavorable prognostic significance of UGT2B17 expression in CLL patients (above) (8, 9), high UGT2B17 expression was associated with enhanced proliferation by 1.7 (*P* < 0.05) and 2.0-fold (*P* < 0.01) for MEC1 and JVM2, respectively (Figures 1E,F). A significantly shorter doubling time for high UGT2B17 expressing cells was evidenced in cell lines by cell counts, MTS assays, and CFSE labeling (Figures 1E–I). No significant difference in cell viability was noted by Annexin V/PI and trypan blue exclusion assays when cells were grown in either basal or serum starvation conditions (data not shown). Robustness of cellular phenotypes was measured periodically for several months to confirm stability of UGT2B17 cell models.

### UGT2B17 Modulates Expression of Genes Related to Prostanoids in Cell Models and Leukemic Cells From Patients

**Figure 2:**
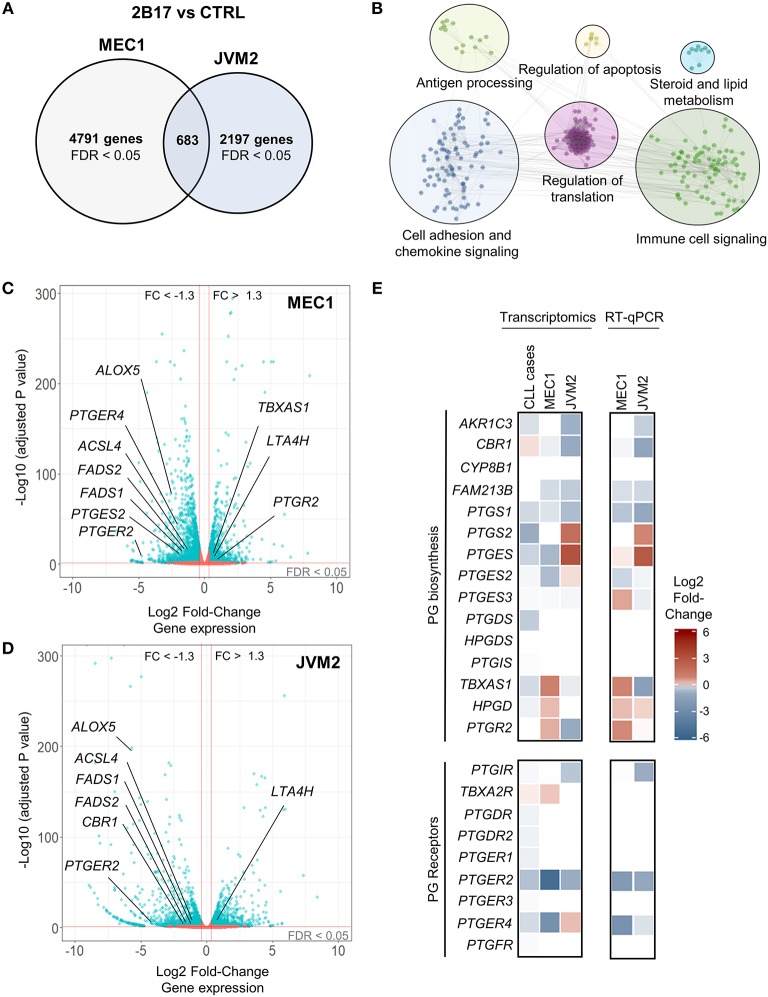
UGT2B17 alters the gene expression profile of lymphoid cells. **(A)** Genes significantly differentially expressed in MEC1 and JVM2 leukemic cells with high UGT2B17 expression vs. controls (false discovery rate (FDR) < 0.05) are represented in the Venn diagram, with 683 genes showing overlapping and concordant changes in both cell lines. Differential expression was established by DEseq2 and EdgeR analyses of gene expression profiles in each cell model determined by RNA sequencing (GSE121626). **(B)** Pathway enrichment analysis of the 683 differentially expressed genes common to both MEC1 and JVM2 models based on the Reactome, KEGG and GO biological process databases. **(C,D)** Volcano plot overviews of genes differentially expressed in high UGT2B17 vs. control MEC1 and JVM2 cells. Blue data points represent significantly (FDR < 0.05) and differentially expressed genes (fold change (FC) > 1.3). Several genes related to eicosanoid biosynthesis and signaling are highlighted. **(E)** left: Differential expression of genes related to prostaglandin (PG) biosynthesis and signaling in UGT2B17-high vs. control MEC1 and JVM2 cells, and in B-cells of CLL patients (*n* = 448, GSE13159) dichotomized into high vs. low *UGT2B17* expression subsets based on median expression level. Gene sets included are shown as defined in the Reactome pathway database. Right: RT-qPCR validation of altered expression in selected genes related to PGE_2_. Data represents mean ± SEM of three independent experiments.

RNA sequencing revealed a strong effect of elevated UGT2B17 on global gene expression. A total of 5474 and 2880 genes were differentially expressed in MEC1-2B17 and JVM2-2B17 cells, respectively, when compared to control cells (*FDR* < 0.05) (Figure 2A). Of those, 683 modulated genes were concordant between models, among which 272 had an absolute fold-change (FC) > 1.3 (*FDR* < 0.05) in UGT2B17 overexpressing cells (Table S3). Pathway analyses using the Reactome and KEGG databases revealed biological pathways overrepresented among common modulated genes such as cell adhesion, chemokine signaling, antigen processing, translation, apoptosis, antigen-receptor signaling as well as steroid and lipid signaling pathways (Figure 2B). The latter was well-represented with 54 genes connected to the metabolism and biosynthesis of lipids or eicosanoids (prostanoids and leukotrienes) significantly modified by UGT2B17 (Table S4). This is plausible given that several bioactive lipids are glucuronidated by the UGT pathway (30). This set of genes was selected for further investigation. Genes involved in arachidonic acid metabolism such as *FADS1, FADS2*, and *ACSL4* were significantly down-regulated in both cell models (Figures 2C,D; Table S4). The leukotriene biosynthesis genes *ALOX5* and *LTA4H* as well as genes from prostaglandin-related pathways were also differentially expressed. This included lower expression of the genes encoding the prostaglandin (PG) E receptors *PTGER2* and *PTGER4*, higher expression of the PG-inactivating enzyme *PTGR2*, as well as decreased expression of the prostanoid biosynthesis enzymes *PTGES2* and *CBR1*. Quantitative PCR confirmed these observations (Figure 2E). Their clinical relevance was supported by the altered expression of these genes in CLL patients expressing high levels of *UGT2B17* in a cohort of 448 cases (GSE13159) dichotomized on the basis of median *UGT2B17* expression (Figure 2E). Notably, genes of the PG biosynthesis pathway and PG receptors were significantly down-regulated in CLL patients with high *UGT2B17* expression (Figure 2E). Also, the expression of genes coding for membrane transporters known to mediate PG influx such as *SLCO3A1* and *SLCO4A1* was reduced in CLL patients with high *UGT2B17*, while the PG efflux transporter gene *ABCC4* was enhanced (not shown). Pathway enrichment analysis of genes commonly altered in both MEC1 and JVM2 further revealed that response to PGE_2_ was a significantly enriched pathway (FDR = 0.032). According to these observations coherent for CLL patients and cell models, the effect of PGE_2_ on B-cell phenotypes and the influence of UGT2B17 expression were investigated further.

### PGE_2_ Increases Cell Death and Inhibits Migration of Primary CLL Patient Cells

**Figure 3:**
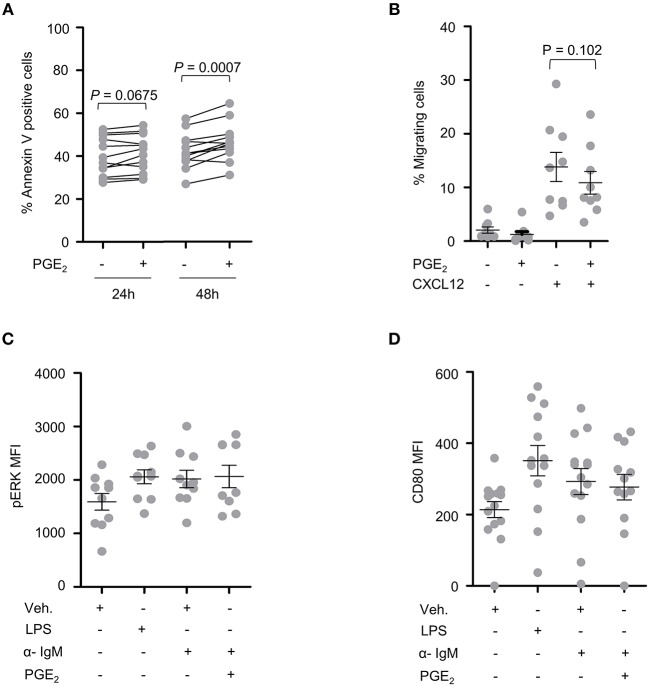
CLL patient cells are sensitive to PGE_2._ **(A)** Cell death of CLL patient PBMCs (*n* = 12) induced by PGE_2_ (5 μM). **(B)** Influence of PGE_2_ (5 μM) on CXCL12-induced migration of CLL patient PBMCs (*n* = 9). Migration assays were conducted 24 h after treatment with PGE_2_. **(C,D)** Influence of PGE_2_ (5 μM) on the activation of CLL PBMCs (*n* = 9) with *F*(ab')_2_ anti-human IgM. Activation was assessed by mean fluorescence intensity (MFI) of **(C)** pERK 15 min after stimulations and **(D)** by CD80 expression 24 h post-treatment. Stimulated cells were primed with PMA 1 h prior to addition of anti-human IgM. LPS was used as a positive control. Purity of CLL fraction was 70% on average, as determined by CD5, CD19, and CD45 expression. In **(B–D)**, data represent means ± SEM of patient samples. Statistics were calculated using a paired-sample *T*-test. Comparisons without reported *P*-value are non-significant (n.s).

PBMCs from 15 CLL patients were analyzed following treatment with PGE_2_ for potential differences in cell death, activation and migration by flow cytometry. Sample purity was assessed by analysis of CD5, CD19, and CD45 expression. A trend toward increased cell death was observed for primary CLL cells treated for 24 h with 5 μM PGE_2_ (*P* = 0.067). By 48 h of treatment, cell death was significantly enhanced by PGE_2_ (*P* = 0.0007), suggesting that PGE_2_ promotes cell death of CLL cells (Figure 3A). CLL cells showed a trend for inhibition of CXCL12-directed migration by 5 μM PGE_2_ when compared to cells treated only with CXCL12 (Figure 3B; *P* = 0.102). PGE_2_ did not alter the activation of PBMCs triggered by IgM stimulation, which was assessed with CD80 and pERK markers (Figures 3C,D). Based on these data, potential anti-oncogenic effects of PGE_2_ and possible interactions with UGT2B17 were investigated further using *in vitro* cell models of B-cell malignancies.

### High UGT2B17 Expression Hinders PGE_2_-Mediated Growth Inhibition in Cell Models

**Figure 4:**
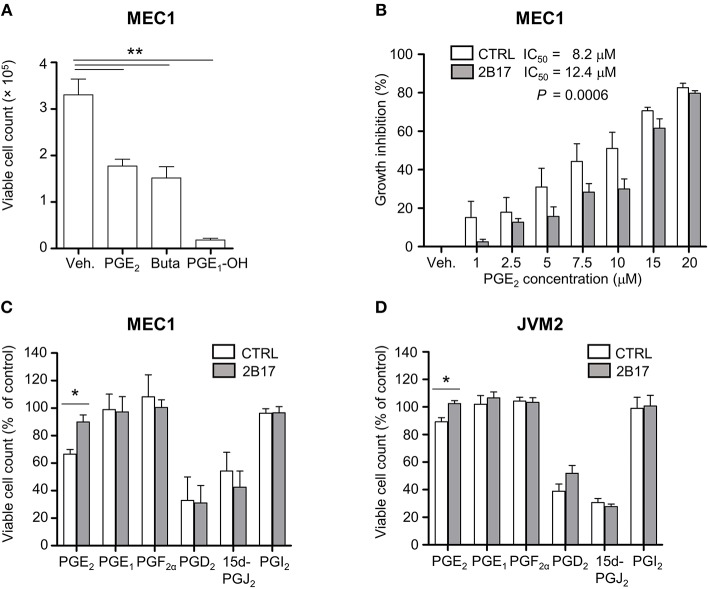
UGT2B17 impedes PGE_2_-mediated inhibition of neoplastic cell growth. **(A)** Influence of PGE_2_ and the EP receptor agonists PGE_1_-OH and Butaprost (Buta) on MEC1 cell growth. Cells were counted 120 h after initiation of treatment with vehicle (Veh) or 10 μM of each compound. **(B)** PGE_2_ IC_50_ was determined in MEC1 cell models by fitting a non-linear curve to normalized response data and comparing the best-fit parameters with the sum of squares *F* test. **(C–D)** Influence of prostaglandins (5 μM) on **(C)** MEC1 and **(D)** JVM2 cell growth relative to vehicle-treated cells, 120 h post-treatment. Data represent mean ± SEM of three independent experiments, each with three technical replicates; ^*^ *P* < 0.05, ^**^ *P* < 0.01. All other data were non-significant (n.s.).

MEC1 cells treated with PGE_2_ or with synthetic agonists of the PGE_2_ receptors EP_2_ (butaprost) or EP_4_ (PGE_1_-OH) showed considerable growth inhibition (Figure 4A). MEC1 cells exposed to increasing physiologically relevant concentrations of PGE_2_ (1–20 μM) further supported a repressive effect of PGE_2_ on B-cell growth, with an inhibition profile significantly different for MEC1-2B17 compared to control cells (Figure 4B). High UGT2B17 significantly decreased the responsiveness of B-cells to PGE_2_, evidenced by a 1.5-fold higher half maximal inhibitory concentration (IC_50_) of 12.4 vs. 8.2 μM for high vs. low expressing cells (*P* = 0.0006). We then tested the effects of other PGs. In these experiments, the level of UGT2B17 expression affected the inhibitory effect of PGE_2_ by up to 33% (*P* ≤ 0.022) but not of the other PGs (Figures 4C,D).

### UGT2B17 Inactivates PGE_2_ and Other Related PGs

**Figure 5:**
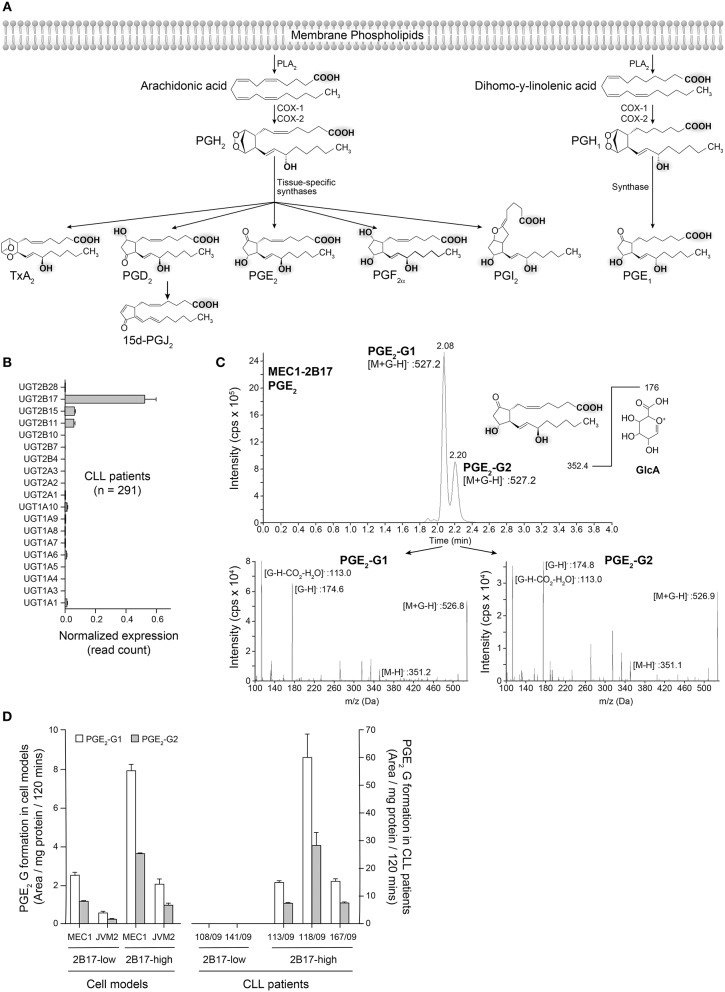
Glucuronidation of PGE_2_ by UGT2B17 and CLL patient cells. **(A)** The prostaglandin biosynthetic pathway. Functional groups on arachidonic acid and related molecules with a potential for glucuronidation are gray-shadowed. PLA2, phospholipase A2; COX-1/-2, cyclooxygenase−1 and−2. **(B)** *UGT2B17* is the predominant UGT gene expressed in CLL PBMCs, based on data from the ICGC CLL cohort. **(C)** Top, Mass spectrometry analysis of PGE_2_ glucuronides (-G) produced by MEC1-2B17 cell homogenates. Elution profiles indicate the formation of two glucuronidated products (PGE_2_-G1 and PGE_2_-G2). The deprotonated PGE_2_ glucuronide ([M+G-H]-) mass (527.2 Da) is consistent with the masses of PGE_2_ (352.4 Da) and glucuronic acid (GlcA; 176 Da). Bottom, The MS/MS fragmentation pattern of each eluted molecule confirmed the identity of PGE_2_-glucuronides with mass/charge (m/z) ratios of fragments specific to PGE_2_ glucuronides, including [M+G-H]^−^, PGE_2_ [M-H]^−^ and the glucuronide moieties, such as [G-H]^−^ and [G-H-CO_2_-H_2_O]^−^. **(D)** LC-MS/MS quantification of PGE_2_ glucuronides formed by PBMC cell homogenates of CLL patient cells and lymphoid cell lines engineered to overexpress UGT2B17. Cells with high (*n* = 3) *UGT2B17* expression efficiently produced the two PGE_2_ glucuronides but not those with low expression (*n* = 2). MEC1-2B17 and JVM2-2B17 cells efficiently conjugating PGE_2_ served as positive controls. Data represents mean ± SEM of at least two assays.

PGs share a lipid backbone and comprise several functional groups (hydroxyl and carboxyl groups) that may be susceptible to conjugation with GlcA by the UGT pathway (Figure 5A). Amongst all 19 human UGT enzymes, UGT2B17 largely predominates in human leukemic B-cells, although with significant variability (CV_UGT2B17_ = 242.9%; *n* = 291 CLL patients) (Figure 5B). We investigated the possibility that PGE_2_ may be a substrate of UGT2B17. The initial series of experiments revealed the formation of two glucuronide (G) derivatives (PGE_2_-G1 and PGE_2_-G2) detected by MS in MRM mode, upon incubations of PGE_2_ with MEC1-2B17 microsomes (Figure 5C). The patterns of fragmentation of individual glucuronides were consistent with the loss of the GlcA moiety (molecular mass of 176) (Figure 5C) and support UGT2B17 targeting two of the functional groups on the PGE_2_ molecule. Enzymatic assays with PGE_2_ further established that primary CLL samples and cell lines expressing high *UGT2B17* levels produced significant amounts of PGE_2_-G1 and PGE_2_-G2 derivatives whereas they were not detected in those expressing low *UGT2B17* levels in CLL patients (Figure 5D).

## Discussion

CLL is a chronic disease characterized by a high degree of heterogeneity, with complex clonal dynamics resulting in an imbalance between B-cell proliferation and death that favors an accumulation of leukemic cells during progressive disease (5). We demonstrated that high UGT2B17 expression is associated with adverse outcome in a cohort of 291 CLL patients, consistent with two independent previous reports (8, 9). UGT2B17 is the only appreciably expressed UGT in CLL cells, although heterogeneously expressed. Our work revealed that high UGT2B17 expression confers a growth advantage to malignant B-cells that is mediated, at least in part, through significant changes in gene expression in PGE_2_-related pathways and its inactivation by the UGT2B17 enzyme. This enhanced proliferation rate results in a significantly larger B-cell population only after several days in culture, consistent with the slow rate of B-cell accumulation in CLL. PGE_2_ emerges as a potential regulator of B-cell growth and apoptosis, a notion supported by previous reports in mouse models and in human B cells (31–36). Similarly, our analysis of primary cells from CLL patients exposed to physiological concentrations of PGE_2_ revealed an enhanced cell death after 48 h and a slight inhibition of CXCL12-mediated migration. Furthermore, in CLL patients' samples expressing high UGT2B17 levels, we also observed PGE_2_ inactivation but not in those with low UGT2B17 levels. One mechanism by which UGT2B17 may modify cancer cell behavior may be through inactivation of PGE_2_, thereby altering homeostatic levels of PGE_2_ and gene expression related to PGE_2_ synthesis, transport and action.

PGE_2_ is a key lipid hormone-like signaling molecule of the eicosanoid family, and plays important roles in disease-associated inflammation and normal physiological functions (37). PGE_2_ is produced from a cascade of enzymatic modifications to arachidonic acid (Figure 5), a polyunsaturated fatty acid esterified in cell membrane phospholipids and a previously reported substrate of UGT2B7 (not expressed in B-cells) leading to its inactivation in the liver (38). Two PGE_2_ glucuronide derivatives were produced by UGT2B17 in leukemic cells, consistent with the conjugation of hydroxyl groups that corresponds to the preferred substrate moieties of the enzyme (39), whereas the hepatic UGT2B7 enzyme may preferentially target the carboxyl group (40). Analysis of PGE_2_-G by nuclear magnetic resonance will be required to confirm this reasoning.

PGE_2_ displays tumor suppressor functions in B-cells and other immune cells by impairing BCR activation and cell proliferation, as well as by triggering cell death (31, 35). PGE_2_ signals through different types of E-series PG receptors (EPs), namely EP_1_, EP_2_, EP_3_, and EP_4_ (41). Past studies in murine cells have demonstrated inhibition of B-cell activation by PGE_2_ through binding and activation of EP_2_ and EP_4_ (31, 32, 36, 42). EP_4_ was shown to be a negative feedback regulator of B-cell activation via the BCR signaling cascade, which is an integral pathway in CLL (31). Our findings that PGE_2_ receptor agonists mimicked the growth-inhibition induced by PGE_2_ support a receptor-mediated mechanism of action in human neoplastic B-cells. The expression of genes related to PGE_2_ signaling, including the EP receptors, was reduced in CLL patient cells and B-cell models with high UGT2B17 expression, likely further disrupting PGE_2_ homeostasis. The mechanism by which UGT2B17 modulates PGE_2_-related gene expression remains to be addressed, but likely involves regulatory PGE_2_ feedback loops. Together, this altered expression profile combined with the direct PGE_2_ inactivation by UGT2B17 support the notion that CLL cells with high UGT2B17 are less responsive to PGE_2_, thus conferring a proliferative advantage that could influence CLL disease course.

Our results provide a first potential mechanism for understanding the role UGT2B17 in CLL by circumventing the actions of endogenous signaling factors such as PGE_2_. This study does have limitations, namely the use of two cell models imperfectly representing CLL, the variability in CLL sample purity and limited number of available patients to investigate the correlation between UGT2B17 expression levels and PGE_2_ response in detail. UGT2B17-dependent glucuronidation of additional endogenous effectors remain to be identified and may contribute to divergent expression profiles and phenotypes reported here for low and high UGT2B17 expressing B-cells.

In conclusion, our findings imply a relevant anti-oncogenic function of PGE_2_ in CLL cells that is blocked by high UGT2B17 expression, through a direct metabolic inactivation of PGE_2_ and altered PGE_2_-related gene expression leading to PGE_2_ unresponsiveness.

## Data Availability

The datasets generated during the current study are available in the Gene Expression Omnibus repository with the accession number GSE121626.

## Ethics Statement

All subjects gave written informed consent in accordance with the Helsinki Declaration and the study was evaluated and approved by local Ethical Research Committees of the Medical University of Vienna (Ethics vote 1499/2015) and the Center Hospitalier Universitaire (CHU) de Québec (A14-10-1205).

## Author Contributions

EA, LV, PC, and VT performed the experiments. EA, MR, LV, EL, and CG analyzed the data. EA performed bioinformatics analyses. EA, MR, EL, and CG designed the research. TL and KV provided patient samples, collected clinical data, and revised the manuscript. EA, MR, and CG wrote the manuscript.

### Conflict of Interest Statement

The authors declare that the research was conducted in the absence of any commercial or financial relationships that could be construed as a potential conflict of interest.
