# Supplementary material for: Inactivation of Prostaglandin E2 as a Mechanism for UGT2B17-Mediated Adverse Effects in Chronic Lymphocytic Leukemia
Source: Front Oncol. 2019 Jul 4;9:606. doi: 10.3389/fonc.2019.00606 (PMC6621974; doi:10.3389/fonc.2019.00606)
Supplement: Supplementary file 1 [file Table_1.docx]

| **Feature** | **N (%)** |
| --- | --- |
| **Age (Median)** | 63.5 years |
|  |  |
| **Sex** |  |
| Male | 12 (80) |
| Female | 3 (20) |
|  |  |
| **Binet B/C** | 2 (13) |
|  |  |
| **Cytogenetics** |  |
| IGHV UM | 7 (47) |
| Del 13q | 5 (33) |
| Del 11q | 1 (6) |
| Del 17p | 0 (0) |

Table S1: CLL patient features
