# Supplementary material for: Inactivation of Prostaglandin E2 as a Mechanism for UGT2B17-Mediated Adverse Effects in Chronic Lymphocytic Leukemia
Source: Front Oncol. 2019 Jul 4;9:606. doi: 10.3389/fonc.2019.00606 (PMC6621974; doi:10.3389/fonc.2019.00606)
Supplement: Supplementary file 2 [file Table_2.docx]

Table S2: Primers sequences and gene description

| **Gene Symbol** | **Description** | **GenBank** | **size (bp)** | **Primer sequence 5'→3'**  **F/R** |
| --- | --- | --- | --- | --- |
| **AKR1C3** | Homo sapiens aldo-keto reductase family 1 member C3 (AKR1C3) | NM_003739 | 120 | CAACCAGGTAGAATGTCATCCGTAT/ACCCATCGTTTGTCTCGTTGA |
| **CBR1** | Homo sapiens carbonyl reductase 1 (CBR1) | NM_001757 | 108 | AGCGCATACGGGGTGACGAA/GCAGCAGGCATTCAGGAGGAT |
| **FAM213B** | Homo sapiens peroxiredoxin like 2B (PRXL2B) | NM_001195736 | 119 | GTCCAGAAGTCCCCAGGCGA/CGCCTCCCTCACACCTCTCTG |
| **PTGS1** | Homo sapiens prostaglandin-endoperoxide synthase 1 (PTGS1) | NM_001271368 | 123 | AGGGAAGAAGCAGTTGCCAGATG/TGGGTGAAGTGTTGTGCAAAGAAG |
| **PTGS2** | Homo sapiens prostaglandin-endoperoxide synthase 2 (PTGS2) | NM_000963 | 175 | ATGGGTAATGTTATATGTTCTCCTGC/TGGTGACTGTTTTAATGAGCTCTG |
| **PTGES** | Homo sapiens prostaglandin E synthase (PTGES) | NM_004878 | 164 | GGCTATACCTGGGGACTTGATG/CAGGAATCCAAGGGGCTAAGA |
| **PTGES2** | Homo sapiens prostaglandin E synthase 2 (PTGES2) | NM_025072 | 172 | AGCCTTCCTCGACTTCCATGC/GGTCTTGAGGGCGCTGATGAT |
| **PTGES3** | Homo sapiens prostaglandin E synthase 3 (cytosolic) (PTGES3) | NM_006601 | 251 | CAAGCATAAAAGAACGGACAGATCA/AATCATCATCTGCTCCATCTACTTC |
| **TBXAS1** | Homo sapiens thromboxane A synthase 1 (TBXAS1) | NM_001130966 | 139 | AGCCAGACATGATCAAGCAGGTG/GCACCTCTGACCTCTTCCCATCT |
| **HPGD** | Homo sapiens 15-hydroxyprostaglandin dehydrogenase (HPGD) | NM_000860 | 201 | GGTAGCGCTGGTGGATTGGAA/TTCTCATTATTCACTCCAGCATTATTG |
| **PTGR2** | Homo sapiens prostaglandin reductase 2 (PTGR2) | NM_152444 | 223 | ACACCTTTCATATTTTCTTGGAGCTA/TGAGGTCAAGAGGATGCATTTC |
| **PTGIR** | Homo sapiens prostaglandin I2 receptor (PTGIR) | NM_000960 | 87 | CAGCAGTGAGATGGGGGACCT/TTGCGGAAAAGGATGAAGACCC |
| **TBXA2R** | Homo sapiens thromboxane A2 receptor (TBXA2R) | NM_001060 | 90 | GTCTACCACGGGCAGGAGGC/GGCCACCACCATGATCCCCA |
| **PTGER2** | Homo sapiens prostaglandin E receptor 2 (PTGER2) | NM_000956 | 153 | AAGAACACAAGATGCAACACAAACT/CACACTGTTTTCATTTCTCCAAGG |
| **PTGER4** | Homo sapiens prostaglandin E receptor 4 (PTGER4) | NM_000958 | 157 | CAGCAGTACATCTCAGACCCTCCT/TGAGGTCTCTGATATTCGCAAAGTC |
| **Hprt1** | Homo sapiens hypoxanthine phosphoribosyltransferase 1 (HPRT1) | NM_000194 | 157 | AGTTCTGTGGCCATCTGCTTAGTAG/AAACAACAATCCGCCCAAAGG |
| **B2M** | Homo sapiens beta-2-microglobulin (B2M) | NM_004048 | 167 | GGGTTTCATCCATCCGACATTG/TGGTTCACACGGCAGGCATACT |
| **UBC** | Homo sapiens ubiquitin C (UBC) | NM_021009 | 127 | CTCGGCCTTAGAACCCCAGTA/AGAATCGCCGAGAAGGGACTAC |
| **ADNg** | Homo sapiens 3-beta-hydroxysteroid dehydrogenase/delta-5-delta-4-isomerase (3-beta-HSD) gene (intron) | M38180 | 260 | GAAGGGCAGAGGTGGAACTAGAA/AACAAAGACCAAAGACCAGTGAGA |

bp: base pairs; F: forward; R: reverse
